# Physical Examination of the Mouth and Throat—The Physical Signs Derivable from the Breath, Lips, Teeth and Mouth

**Published:** 1880-12

**Authors:** G. V. Poore

**Affiliations:** Professor of Medical Jurisprudence, University College; late Assistant Professor of Clinical Medicine; Assistant Physician and Physician in Charge of the Throat Department of the Hospital, etc. Delivered to the Junior Class of Clinical Medicine, University College.


### ARTICLE V.

### *Lectures on the Physical Examination of the Mouth and Throat The Physical Signs Derivable from the Breath, Lips, Teeth and Mouth.*

BY G. V. POORE, M. D., F. R. C. P.

Professor of Medical Jurisprudence, University College; late Assistant Professor of Clinical Medicine; Assistant Physician and Physician in Charge of the Throat Department of the Hospital, etc. Delivered to the Junior Class of Clinical Medicine, University College.

*Gentlemen.*—It is my duty to bring to your notice the various physical signs of disease which are to be obtained from an examination of the throat and windppie; but inasmuch as it is impossible to properly examine the throat without at the same time examining the mouth and nose, I think I shall be best fulfilling my duty by dealing methodically not only with the throat, but also with the oral and nasal cavities which lie above it.

The physical signs met with in these regions of the body appeal not only to the sight, touch and hearing, but occasionly to the sense of smell as well; and the first thing which forces itself on our attention is often the odor of the breath.

The *smell of the breath* is a valuable physical sign, and in many diseases is so characteristic as to enable the man of experience to form a diagnosis from it alone with almost absolute certainty. It is impossible to describe the various odors of the breath: experience alone will enable you to distinguish one from the other, and I shall merely content myself with catalogueing some of the most distinctive of them. The smell of drink is the most common of all, and in case of insensibility is often a valuable indication of the cause. It may give a valuable hint as to the habits of the patient; and I would here remind you that over-indulgence in alcoholic liquors is one of the most common causes of congestion and catarrh, both of the pharynx and larynx. You must not run too quickly to the conclusion that because a man's breath smells of drink he is necessarily a drunkard, for a single glass of wine or beer is sufficient to impart an odor to the breath for some time after it has been taken. When directing attention to the alcoholic smell of the breath in the presence of the patient, I am in the habit of speaking of it as *osnosmia* since patients naturally resent having attention bluntly called to the fact that they smell of drink.

The presence of carious teeth imparts an odor to the breath which is quite characteristic, and which, according to Mr. Salter, resembles no other odor except that given off by the genus of neuropterous insects called Chrysopa. Want of attention to the mouth and allowing food to lie between the teeth and decompose, or the presence of decomposing matters in the crypts of the tunis, imparts a foul odor to the breath. A disordered stomach also causes the breath to be fetid.

A peculiarly disgusting and characteristic odor of the breath is present in those cases of chronic inflammation of the nasal and pharyngeal cavities, which are known from this fact as ozæna, and which are most often due to caries or necrosis of the nasal bones, which is generally of syphilitic origin. The smell, however, may be present without any disease of the bones in case of chronic inflammation of the cavities occuring in scrofulous subjects.

In cases of dilatation of the bronchial tubes accompanied by ulceration and copious purulent discharge, the smell of the breath is peculiar and almost diagnostic of the condition, and in gangrene of the lung the odor of the breath reaches a degree of foulness which, once smelt, can never be forgotton.

In cases of fever with high temperature, a dry mouth, and the accumulation of sordes on the teeth and gums, the smell of the breath is peculiar. In pyæmia and in diabetes the breath has a sweet odor, but the odor in each of these diseases is perfectly distinguishable.

With inflamed gums the breath is apt to smell. This is peculiarly the case in patients under the influence of mercury, and the term mercurial odor of the breath is one in common use. In scurvy, the breath is apt to be very foul. It is needless to say, that certain articles of diet, as garlic and onions, and certain drugs, as turpentine, copaiba, and some of the essential oils, are detectable in the breath.

The *inspection of the lips* is capable of furnishing many facts which are of the greatest service in forming a diagnosis. The *form* of the lips is characteristic in different races; thus, the thick lips of the African negroes and the thin lips of most European races are well known. In conditions of general plethora the lips look swollen and big. A few cases have been recorded of great hypertrophy of the lips and neighboring parts, a notable example being given by Mr. Barnwell in the eighth volume of the "Clinical Society's Transactions."

The *color* of the lips is a matter of great importance. After great loss of blood the lips may appear of a waxy whiteness, and such an appearance should at once lead to questions likely to elucidate this point. A recent confinement attended by hemorrhage is the most common cause of this appearance in women. Anæmia and leucocythæmia, arising from no matter what cause, produce a pallor of the lips, and in investigating cases of anæmia, we invariably look to the mucus surface of these parts. It is right to remind you, however, that undoubted evidence of hydræmia may be present without any very obvious alterations of the tint of the lips.

The lips are often unduly red in cases of general plethora and in the early stages of many febrile conditions. A cyanotic tint of the lips may be due to extreme cold, to those malformations of the heart which gives rise to the condition known as cyanosis, and to mal-æration of the blood arising from no matter what cause, atmospheric, pulmonary, or cardiac. A patch of herbs on the lips (herpes labialis) is very commonly seen. It is a common accompaniment of an ordinary cold, and it is well to bear in mind that such an appearance may be indicative of more serious trouble, such as pneumonia. It is sufficiently often an accompaniment of pneumonia to make it incumbent upon us always to investigate this point when we are confronted with a patch of herpes on the lips.

In febrile conditions the lips get dry and cracked, and sordes accumulate upon them. Sordes are collections of dried mucous, evaporated saliva, and food particles which cannot be removed, owing to the general dryness of the mouth and the paucity of the salivary secretions. This condition of the lips is seen in the most extreme degree in the state known as the typhoid condition, in which also the lips are often brown or almost black.

Round the margins of the lips are occasionally seen cracks white lines, and little pits, the latter reminding one of the appearance known as the lineæ albicantes which occur on the abdomen after pregnancy. These appearances occurring on the lips are sufficient to raise a suspicion of congenital syphilis. The other indications of syphilis which we may find upon the lips are: 1. A true infecting sore or hard chancre, which is happily rare; and, 2. Mucous tubercles, which may be present in cases of congenital or acquired syphilis. These mucous tubercles have the same appearance when seen here as when seen seen elsewhere—flat, slightly elevated patches, with a dirty-whitish surface, surrounded by a congested areola. Epithelioma is among the more rare diseases of the lips, concerning which one one should be on one's guard.

The *movement* of the lips is a matter of great diagnostic importance. The muscular power of the lips may be impaired or abolished in several distinct conditions, such as hemiplegia, facial palsy, bulbur or labio-glosso-laryngeal paralysis, and general paralysis of the insane.

In hemiplegia the lip palsy is often slight, and in very slight cases which have partially recovered, a trifling drooping of the prolabium of the upper lip on one side, just sufficient to destroy the symmetry of the "Cupid's bow," is all that we can detect. The observation of this slight dooping and want of symmetry should always lead to an investigation into the history of the patient, and to questions likely to elucidate the question of hemiplegia. In marked cases of hemiplegia, and in cases of facial palsy from disease or injury to the trunk of the facial nerve, the paralysis of one-half of the lips is easily demonstrated, and on asking the patient to show the teeth it will be observed that the teeth are imperfectly exposed on the paralyzed side, and the angle of the mouth is drawn over to the sound side. Facial palsy may be double, and then this want of symmetry is not observed, but the face is expressionless, and the teeth and gums cannot be exposed.

In bulbar paralysis the condition is usually bilateral, and the patient is quite unable to move the lips. In the latter stages of this disease the lips waste, and the under lip droops so as to expose the gums and allow the saliva to run out of the mouth.

In general paralysis of the insane there is a paretic condition of the lips, and when they move they do so in a hesitating, jerky manner, which is very characteristic.

In alcoholism the movement of the lips is also often tremulous. In chorea the lips are liable to those uncertain, jerky movements which are so characteristic of this condition. In "muscular tie" one side of the mouth may be the seat of spasmodic movement. Lastly, in tetanus spinal meningitis there occurs that condition which is called the risus sardonicus, which is caused by a spasmodic retraction of the angles of the mouth.

*Dribbling of saliva* is a symptom which is due to many causes. It may be due to an excessive secretion of saliva, a condition seen in cases of mercurial poisoning and in some other states. It is present in cases where there is deficient movement of the lips and tongue, as in bulbar paralysis, or in cases where the movement of the tongue is rendered impossible or painful by the presence of sores and ulcers. In patients also with whom the act of swallowing is impaired or painful, as in cases of paralysis or stricture of the pharynx, or inflammation of the tonsils or throat, dribbling of the saliva is apt to occur. In children dribbling is a physiological condition, owing to a want of vigor and purpose in the movement of their lips and tongues, and in idiots this infantile condition would seem to be permanent. Old writers consider the dribbling of saliva to be characteristic of idiots and madmen.

An *inspection of the gums* occasionally affords important evidence of disease. Their *collor*, like the color of the lips, may be pale, or red, or livid, and is an indication of anæmie,or plethora, or those conditions mentioned in connection with the lips which give rise to a cyanotic tint. The gums are sometimes spongy and congested and liable to bleed at slight causes. This is often the case in depressed conditions of health, arising from whatever cause. It is present in a marked degree in persons who are under the influence of mercury, and to a less extent in those who are taking iodide of potassium. In leucocythæmia and in Hodgkin's diseases the gums are often swollen and pale, and occasionally they are stated to become gangrenous. In purpura, hemorrhage from the gums is a common occurrence. In scurvy the gums are very greatly and remarkably affected. They become sore and apt to bleed at the slightest touch, and get swollen, spongy, and livid. The lividity is stated to be most marked at the free edges. The swelling of the gums is so great as occasionally to obscure the teeth, and in extreme cases they protrude between the lips. They get livid and almost black, and undergo sloughing and ulceration, which causes the breath to be peculiarly offensive. The sloughing may leave the roots of the teeth exposed, and in such cases the teeth commonly fall out. Dr. Buzzard states that this condition of the gums is by no means invariably present in scurvy, and that all the other symptoms of the disease may be present in a marked degree while the gums are not noticeably affected. Indeed, the gums in scurvy may occasionally be paler than usual and contracted.

A blue line upon the gum may, in the vast majority of cases, be taken as certain evidence that the patient is suffering to a greater or less extent from lead-poisoning. This "blue line" is due to a deposit of lead sulphide in the tissues of the gums. Dr. Hilton Flagge has made sections of the margin of a gum affected with a lead line, and by the aid of the lower powers of the microscope was able to see that the discoloration was not uniform, but was distributed in the form of rounded loops. The pigmentation was seen to be due to minute granules, and these granules were situated sometimes in the interior of the smaller blood-vessels, and sometimes outside them in the tissue immediately adjacent. The deposit is in reality black, its blue appearance being due to the fact that it is seen through a thin translucent layer of gum. Care must be taken not to mistake the purple congested edge of the gum of persons who do not clean their teeth for the deep blue line which is caused by lead. The blue line is produced by the action of hydrogen sulphide upon the lead which is presumably circulating in the blood. The hydrogen sulphide is produced by the decomposition of food particles lodging between the teeth and adhering to the tartar. Persons who are careful to keep the teeth clean, and in whom no decomposition of the food particles takes place, may be suffering from lead-poisoning and yet have no lead-line upon the gums. The lead-line once formed, and being due to the deposit of an insoluble salt, may remain for months after the system has been freed from lead. Persons who have been exposed to the action of lead may exhibit no line upon the gums until after the administration of iodide of potassium. This is difficult of explanation, but the fact admits of little doubt. The blue or black discoloration caused by lead is not always limited to the margin of the gums, but may occasionally form black patches on the inside of the lips or cheeks.

Occasionally among the ill-fed and dirtily-kept children of the poor, and especially during the first dentition, the gums become swollen and the edges ulcerate, the ulcerated surface being covered with a dirty-gray secretion. This condition is known as gingivitis, accompanied by offensive breath and some increase in the flow of saliva.

The *teeth* often afford valuable evidence of constitutional conditions. Delayed dentition is apt to occur in children that are debilitated from any cause, but more particularly in the case in rickets. Finding the dentition delayed, we should always search for either evidence of rickets.

The milk teeth should begin to appear at the seventh month, and should all be "cut" by the end of the second year. The teeth appear in the following order—central incisors, lateral incisors, anterior molars, canines and posterior molars; and each of these five groups appears by the seventh, ninth, twelfth eighteenth and twenty-fourth month; the number of teeth which a child should have at the end of the months named being four, eight, twelve, sixteen, twenty. It may be some help to the memory to call attention to the fact that when a child is twelve months old there should be twelve teeth in the mouth. These numbers are liable to great deviation even in health. Some healthy children are precocious, while others are backward in the matter of dentition. The teeth may be wholly or in part deficient as the result of congenital defect. Caries or decay of the teeth is so common in this country that very few escape from it. It is more common in women than in men, and is predisposed to by pregnancy and by the scrofulous and tuberculous constitutions. It is said to be caused by the generation of acid from the fermentation of food particles lodged between the teeth. There is a condition known as "rocky" enamel, in which the enamel of the teeth is grooved and pitted and honeycombed. This condition is brought about by rickets, or by any depressing illness occuring during dentition. Occasionally the teeth get excessively worn, so that they appear truncated, and the dental arch presents the appearance of a flat level border, the exposed dentine presenting a yellowish appearance. This condition, of which a very good example was lately attending in my out-patient room, is rare, and is said to be predisposed to by syphilis, and to be favored by the use of gritty food. Mr. Jonathan Hutchinson has pointed out that a peculiar condition of the permanent teeth often exists in patients who are the subjects of inherited syphilis. The incisors and canine teeth are small, peg-like in shape, narrow at the free edge, and either excavated by a crescentic notch at the margin or marked by a crescentic groove. The conical condition is most marked, according to Salter, in the lower, and the crescentic notch is most conspicuous in the upper incisors. When the teeth are lost very early in life, inquiry should always be made as to whether the patient has taken much mercury, and if so, for what reason.

The *mucous membrane of the mouth* is sometimes swollen and red as part of a general catarrh. It may be swollen in consequence of gastric irritation brought about by errors in diet. In children who are ill fed, and especially during dentition, small, circular, painful ulcers called apthæ very frequently appear upon the gums and the internal surface of the cheeks. They are almost always an indication of gastric disturbance from injudicious feeding. When we get the mucous membrane of the mouth inflamed, and upon the inflamed surface a parasite funbus (the oidium albicans) growing, we have the well known disease called thrush. The mouth, tongue, and palate, and pharynx may be covered with white patches, and we may be in doubt whether these patches are due to curdled milk or diphtheria, but if a small quantity be placed under the microscope with a drop of caustic potash, the well known mycelium and spores of the oidium albicans are easily seen, and serve to clear up all doubts. Whether the fungus is the cause of the inflamed condition, or whether the inflamed patches form a fitting nidus for the growth of the fungus, is an open question. Thrush never occurs in well nursed children, and if a young child is fed upon gook milk and nothing else, thrush seldom appears. When, however, mothers give farinaceous matter to very young children, often combined with milk which is slightly sour, this sticky mixture adheres to the inside of the mouth, and if the mouth be not very carefully cleansed out after every meal, the decomposing food particles irritate the mouth, cause it to inflame, and form a soil upon which the oidium grows luxuriantly.

Thrush is liable to occur in adults towards the termination of chronic illnesses, when they are too weak to cleanse their mouths by vigorous movements of the tongue. I have seen patches of thrush also occurring in a patient the subject of labio-glosso-laryngeal paralysis, because the movements of the mouth were too feeble for the purpose of properly cleansing it. The lesson to be learnt from these facts is that in feeble persons the mouth needs to be artificially cleansed after feeding, by being sponged out with some antiseptic, such as a solution of borax, or, perhaps, there is nothing better than peppermint water, which to many persons is agreeable and refreshing. —*British Journal of Dental Science.*

